# Various Ways to Be Negative: Biophysical Characterization of Polyanionic Biomolecules

**DOI:** 10.1021/acs.jpcb.5c05329

**Published:** 2025-12-23

**Authors:** Noa Binnes, Ilan Edelstein, Yaakov Levy

**Affiliations:** Department of Chemical and Structural Biology, 34976 Weizmann Institute of Science, Rehovot 76100, Israel

## Abstract

Negatively charged biopolymers (i.e., polyanions) are ubiquitous across all domains of life and participate in a vast array of cellular processes. Their remarkable diversity raises fundamental questions about how their biophysical properties enable such functional breadth. To investigate these relationships, we performed all-atom molecular dynamics simulations of 11 representative polyanions spanning three major classes of polyanionic biomacromolecules: polynucleotides, polypeptides, and polysaccharides. Each polymer was modeled at a fixed length of 30 repeat units but differed in monomer size, charge per monomer, and linear and radial charge density. We systematically examined how these intrinsic features modulate their biophysical properties and influence solvent organization and conformational preferences in mono- and divalent counterion environments. Our analyses reveal that polyanions differ markedly in compactness and flexibility and that their conformational preferences respond in a system-specific manner to cation identity. While some polymers are strongly modulated by sodium or calcium, others remain comparatively insensitive. Collectively, the polyanions span a broad landscape of conformational space defined by their intrinsic features and the resulting biophysical properties, with each macromolecular family occupying a distinct region of this space. Even within a given family, subtle differences in intrinsic features lead chemically related systems to exhibit unique biophysical properties. These findings show that diverse classes of polyanions possess tunable biophysical properties that evolution could exploit to support specific biological functions, and they further highlight the intriguing question of why biological systems tend to favor polyanions over their positively charged counterparts.

## Graphical abstract

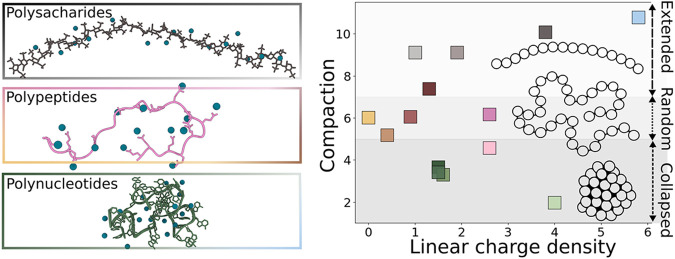

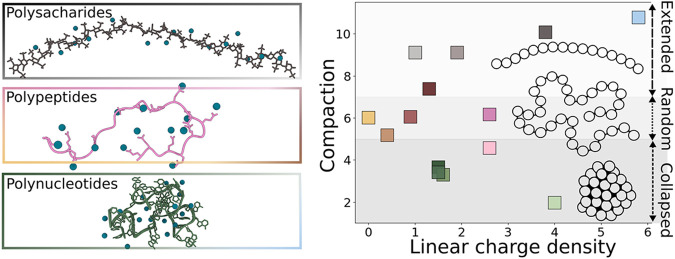

## Introduction

Naturally occurring water-soluble polymers are ubiquitous in known living organisms, with their existence predating eukaryote evolution.^1,2^ The solubility of these polymers is a direct corollary of the presence of numerous groups that are ionizable under physiological conditions, and they can exhibit polyampholytic (i.e., mixed charge) or polyelectrolytic (i.e., uniform charge) characteristics.^3^ When the polyelectrolytes contain functional groups that are negatively charged under physiological conditions, they are called polyanions, whereas when they contain positively charged groups, they are termed polycations.^4,5^ Polyampholytes can vary based on the ratio and arrangement of their positive and negative charges, whereas polyelectrolytes differ primarily in the nature of their monomers, which influences their charge density.

Among the various types of polyelectrolytes, biological polyanions stand out due to their remarkable diversity and widespread presence in living systems when compared with polycations. Native polyanions can be divided, according to the chemical nature of their monomeric building blocks, into polynucleotides, polypeptides, and polysaccharides.^2,6,7^ The functions of these endogenous polyanions vary greatly but are often essential to maintaining and perpetuating life. For example, polyanions constitute both DNA and RNA, and they are thus crucial for enabling transcription and regulating translation. Cellular scaffolding includes key contributors to structural integrity, such as actin microfilaments, microtubules, and viscous gels composed of glycoproteins and polysaccharides.^2,4,5,7^

In contrast to the wide variety of biological polyanions, natural polycations are rare, with only a few examples known. These include the polysaccharide chitosan and basic amino acid repeats of lysine (Lys, K) and arginine (Arg, R).^5,8–11^ Among the polysaccharides, chitosan is found in fungal cell walls and has been thoroughly studied for its uniquely cationic nature.^5,8,9^ Polycations formed from basic amino acids appear as repeat units in proteins but, with a maximum length of 10 residues, they are shorter and far rarer than the repeats formed by their acidic counterparts, glutamic acid (Glu, E) and aspartic acid (Asp, D).^10^ Polycations exist primarily in intrinsically disordered regions (IDRs) of proteins, such as tau and growth factors, which most commonly interact with polyanions.^10,11^ Polycations have been shown to mediate protein–protein interactions and to disrupt cell membranes to instigate polynucleotide transfection, polyanion transportation, or cell death via apoptosis, autophagy, or necrosis.^11–13^

Polyanions participate in a wide range of biological processes and are also linked to both disease progression and therapeutic applications.^2,14–17^ Their ability to interact with a diverse array of biomolecules can be driven by various factors, such as their charge, which facilitates both nonspecific electrostatic interactions that act over long distances and specific interactions, such as hydrogen bonding and van der Waals forces, which act over shorter distances.^2,4,5^ These electrostatic interactions can enhance association rates (e.g., between proteins or between proteins and DNA) by up to 2 orders of magnitude, even after accounting for dielectric properties and ionic screening in physiological solutions.^18^ Processes mediated by these interactions include proper protein folding and the assembly of polyanions, rapid protein binding or expulsion from charged areas (which even enables polyanions to function like chaperones under some circumstances), and regulation of catalytic activity.^19,20^

One example of the role of polyanions in biological processes is their involvement in neurodegenerative diseases through their interactions with tau protein.^21–26^ Tau, an intrinsically disordered protein (IDP) with positively charged regions, associates with microtubules to promote self-assembly.^21–26^ Polyanions, such as polysaccharides and polyphosphates, drive tau aggregation and amyloid fibrillation, which are implicated in neurodegenerative disorders, but their polymeric properties—such as charge density and valency—likely determine the specific outcomes, including aggregation kinetics and fibril morphology.^24^ This highlights two crucial aspects: first, that charge is central to the activity of polyanions and their partner biomolecules, and second, that variations in polyanions’ polymeric properties lead to different biological outcomes. Although charge is the common driving force, it is the specific properties of each polyanion that shape the kinetics and dynamics of the interactions.^24^ The influence of post-translational modifications (PTMs) on tau similarly underscores this concept, as they too alter tau’s behavior, leading to distinct aggregation and fibrillation outcomes depending on charge interactions.^27^ Additionally, polycations can inhibit this aggregation, further highlighting the central role of electrostatics in these processes.^21^

Given the abundance and versatile roles played by polyanions in native processes, we focused on examining how the different charge distributions and structural diversity of biological polyanions influence their biophysical properties in the presence of monovalent versus divalent cations. This multisystem approach aims to explore the shared features of negative charge across polyanions, enabling comparisons within and between the three polyanion categories of interest, and to reveal specific structural adaptations in response to distinct ionic environments. We selected 11 diverse representative systems of polyanions from each of the three classifications: polynucleotides (Figure 1A), polypeptides (Figure 1B and C), and polysaccharides (Figure 1D).

**Figure 1:**
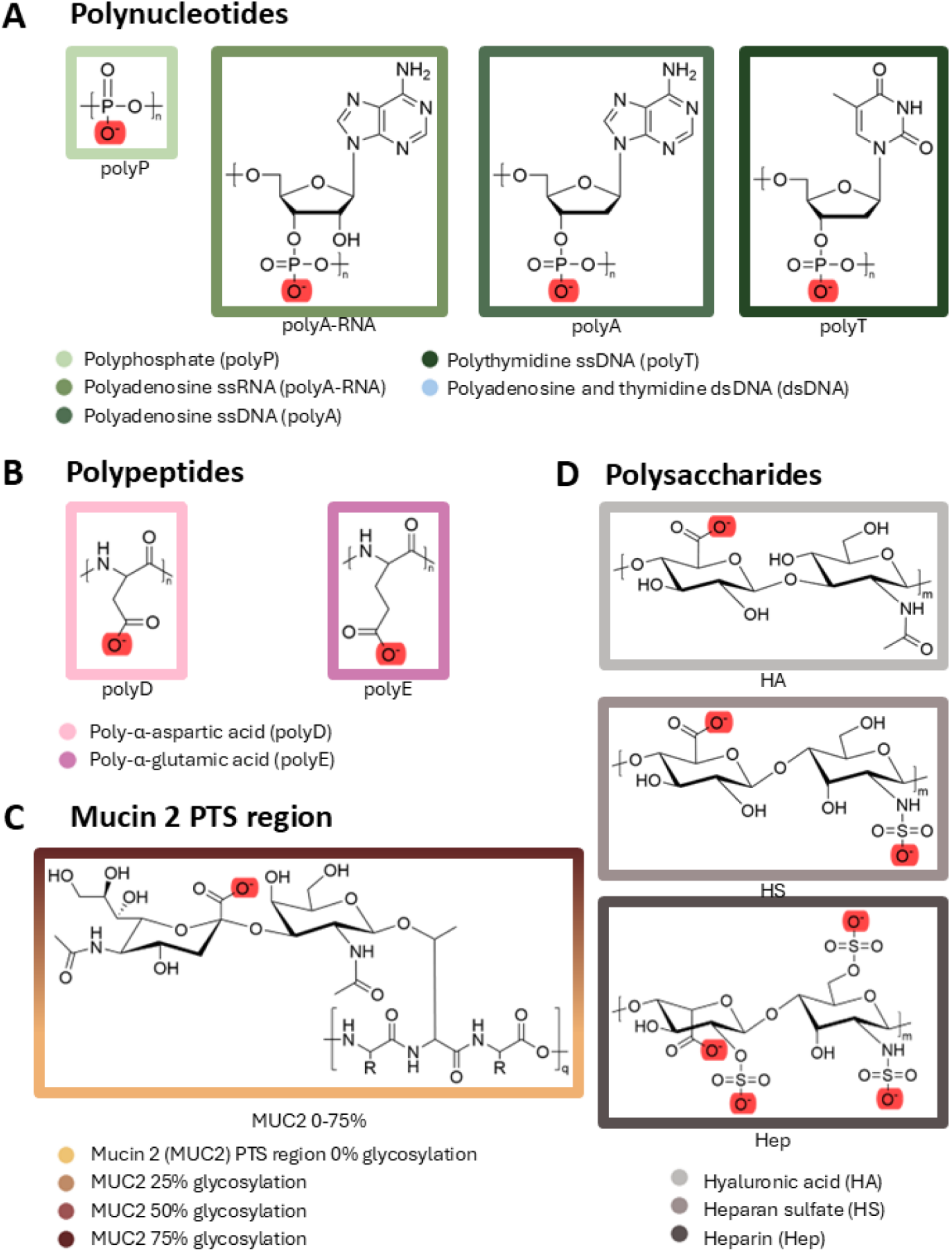
Building blocks of polyanions. (A) Polynucleotides: polyP, polyA-RNA, polyA, polyT (to study ssDNA and dsDNA). (B) Polypeptides: polyD and polyE. (C) MUC2, glycosylated to four different extents (0–75% glycosylation). (D) Polysaccharides: HA, HS, and Hep. Acidic oxygens are highlighted in red, although they are usually in resonance. The full names of the polyanions and matching border colors are set out in the key.

**Polyanionic polynucleotides** include endogenous polyanions, such as double-stranded DNA (dsDNA), single-stranded DNA (ssDNA), and single-stranded RNA (ssRNA), which possess some of the strongest charges among known biopolyelectrolytes due to the charged orthophosphate groups (PO_4_^3–^ or P for short) on their backbones (Figure 1A).^28^ The ubiquitous presence of DNA and RNA across all domains of life suggests that their strong negative charge plays an essential role in their stability, structural flexibility, and ability to regulate key processes, such as gene expression and chromatin condensation through interactions with counterions. Inorganic polyphosphate (polyP; Figure 1A) features a backbone resembling that of nucleotides, with negatively charged orthophosphates covalently linked via highly energetic phosphoanhydride bonds.^29–33^ This linear, unbranched polymer can range in length from three to thousands of subunits, and its presence has been detected in virtually all tested lifeforms, from prokaryotes to eukaryotes.^29,31,33,34^ In bacteria, polyP’s strong negative charge aids in stress resistance, enabling it to function as a chaperone and scaffold for proteins (e.g., in biofilm construction), thus contributing to bacterial virulence.^31,35,36^ In eukaryotes, polyP’s negative charge plays a crucial role in blood coagulation, and during vertebrate bone formation, it enables enzymatically regulated calcium release from storage while also serving as a phosphate source.^36–39^

**Polyanionic polypeptides** often correspond to charged IDRs in proteins, many of which are polyanionic, consisting primarily of D and E residues.^3,10^ Such D/E repeats were found to be significantly longer than their polycationic counterparts (comprising Lys and/or Arg), with some extending up to 49 D/E repeats in length.^3,10^ Glutamic acid is the second most common disorder-promoting residue, and both it and aspartic acid have a carboxylic acid functional group on their side chains with p*K*_a_ values of 4.25 and 3.65, respectively, rendering them negatively charged at physiological pH levels.^40–42^ Another way proteins can become negatively charged without containing acidic amino acids is via PTMs, such as phosphorylation or glycosylation with negatively charged glycans. For instance, the proline, threonine, and/or serine (PTS) region of the mucus-forming protein mucin 2 (MUC2; see Figure 1C) is typically extensively glycosylated.^43,44^ This *O*-glycosylation is initiated via the addition of *N*-acetylgalactosamine (GalNAc) to either threonine or serine residues in the PTS region. Its negative charge arises largely from terminal sialic acids (e.g., *N*-acetylneuraminic acid; NeuNAc), which play an important role in cell–cell and cell–pathogen communication.^44,45^ The negatively charged groups in MUC2 allow for its reversible compaction into secretory granules during exocytosis via calcium ion bridging and charge shielding^44^ and contribute to mucus viscosity reduction when the concentration of sodium increases.^44^ Moreover, its anionic potential enables high water retention, thus affording mucin its gel-like properties and allowing mucin to play a critical role in regulating the movement of particles across the mucus barrier.^44,46^

**Polyanionic polysaccharides** (Figure 1D) acquire their negative charges from specific chemical modifications to their component sugars.^45^ For example, uronic acids, such as d-glucuronic acid (GlcA) or l-iduronic acid (IdoA), contribute a negative charge through oxidation of the C6 position to a carboxylic acid.^45^ Additionally, sulfation of hexosamines or uronic acids further increases the negative charge of these chains under physiological conditions.^45^ This characteristic is especially embodied in glycosaminoglycans (GAGs), which are unbranched linear polysaccharides found in most animals and consist of alternating uronic acids and hexosamines comprising thousands of residues.^47^ Hyaluronic acid (HA) is a GAG that stands out because of its reliance solely on the carboxylic acid group for its negative charge.^47,48^ HA plays key roles in water retention and transport, lubrication, and cellular receptor-mediated processes.^47,48^ In contrast, other GAGs, such as heparan sulfate (HS) and heparin (Hep), achieve their high negative charge through additional modifications, namely sulfation. This additional charge makes these similar, yet distinct, molecules some of the most negatively charged GAGs.^49^ The charge variation in HS plays a critical role in its ability to regulate biological processes, such as cell proliferation, development, migration and inflammatory responses,^50^ and the highly negatively charged Hep grants it significant affinity for many proteins possessing positively charged domains, termed heparin-binding proteins.^47^ The charge and structural variability seen in GAGs is an example of how the negative charge of polyanions, and their distinctive polymeric properties can drive their biological activity and structural diversity.

To quantify the compositional diversity of polyanions and elucidate the structural differences among and between various native polyanionic groups, we employed atomistic molecular dynamics (MD) simulations. This method allows for a detailed exploration of the interplay between charge density, backbone flexibility, and interactions with ions and is thus particularly well-suited to the current examination of how these biophysical characteristics can influence the properties of polyanionic biomolecules.

## Methods

### All-Atom Molecular Dynamics Simulations

To investigate the influence of charge distribution and structural variability on the polymeric properties of polyanions, we performed all-atom MD simulations on the 11 selected polyanions (Figure 1), each of which was 30 monomers in length. This length is sufficiently long to capture the conformational preferences of the studied polyanions. These simulations aimed to explore how the molecular properties of each polyanion and its ionic environment affect its biophysical characteristics, thereby enabling a systematic comparison of the structural and charge-driven properties of polyanions.

### Model Preparation

A molecular model was generated for each studied polyanion. PyMol 2.5.2 was used for polypeptides and polyP, whereas Coot 0.9.8.93 was used to generate files for dsDNA, ssDNA, and ssRNA. GAGs and MUC2 (sequence TTTTTVTPTPTPTGTQTPTTTPITTTTTVT) sugar chains were created with the GLYCAM web tool.^51^ GLYCAM sugar names were amended to CHARMM-appropriate sugar names.^52^ For MUC2, varying levels of glycosylation (0%, no sugars; 25%, 5 sugars; 50%, 10 sugars; 75%, 15 sugars) were applied at threonine *O*-glycosylation sites using CHARMM-GUI.^53–58^ This selection of glycosylation sites was based on regions rich in potential glycosylation targets to achieve various glycosylation levels. The glycosylation sites for the 25%, 50%, and 75% glycosylated MUC2 are [2, 5, 15, 24, 28], [2, 5, 7, 9, 13, 15, 21, 24, 25, 28], and [1, 2, 5, 7, 9, 11, 13, 15, 17, 20, 21, 24, 25, 27, 28], respectively. The *O*-glycans were constructed with *N*-acetylgalactosamine (GalNAc) and α(2 → 3)-linked *N*-acetylneuraminic acid (Neu5Ac). For the polynucleotides, dsDNA comprised alternating adenosine and thymidine units; ssDNA contained polyadenosine (polyA) and polythymidine (polyT), and ssRNA was constructed from polyadenosine (polyA-RNA). CHARMM-GUI was also employed for polyA-RNA, polyE, and MUC2 simulation preparations.^53–58^ Phosphate groups at the 5′ end of ssDNA and ssRNA were removed for consistency with GROMACS terminal base definitions, thus adjusting the overall charge of these systems.

### Simulation Setup

Simulations were performed using GROMACS 2022 under consistent conditions across all systems: TIP3P water model, pH 7, temperature maintained at 303.15 K with the Nosé–Hoover thermostat, and 1 bar pressure applied in an isotropic fashion using the Parrinello–Rahman barostat. Each polyanion was simulated in either a cubic or dodecahedral box, with box sizes adjusted according to molecular size (1–2 nm edge distance), except for dsDNA, which was simulated in a 3 nm triclinic box.

For Lennard–Jones interactions, a cutoff distance of 1.2 nm was employed, and for electrostatic forces, the fast Smooth Particle-Mesh Ewald (SPME) method was applied with a 1.2 nm cutoff. Neutrality was preserved by adding counterions (NaCl and CaCl_2_). To maintain a consistent charge balance and explore cation localization and valency effects, the divalent calcium concentration was set to half that of monovalent sodium (0.0625 M Ca^2+^ and 0.125 M Na^+^). Three replicates were performed per cation condition. A CHARMM36m force field was applied to all systems, except for the polyP and dsDNA systems, whose force fields were generated using the AMBER Barcelona 99 force field, with the AMBER force field for polyP being derived from experimental tests.^59^ All simulations were performed over 250 million steps (0.002 ps/step), with data saved every 500 steps, totaling 500 ns per run in all cases. After box construction and solvation, energy minimization was carried out using either the steepest descent or conjugate gradient algorithm, followed by equilibration with the NVT and NPT ensembles. To remove visual jumps caused by periodic boundary conditions, the simulations were processed using the GROMACS command gmx trjconv (sampling every 200 steps), thus ensuring continuous molecular trajectories for analysis.

### Charge Density of the Polyanions

Charge density plays a crucial role in determining the interactions of polyanions with ions and water molecules, directly influencing their biological properties. Two charge density measures are potentially important. The first is the charge density on the monomeric building blocks, which describes charge distribution along the backbone (i.e., along the main *x*-axis, shown in purple in Figure 2A) and is therefore termed the linear charge density, designated λx and computed according to the definition: $\lambda x=\frac{q_{\mathrm{tot}}}{dx\times N}=\frac{q_{\mathrm{tot}}}{L_{c}}$ where *q*_tot_ is the total number of charges in the polyanion, d*x* is the monomer length, *N* is the total number of monomers, and $L_{c}$ is the contour length of the fully extended polyanion. The monomer length is 6.1–6.3 Å for single-stranded polynucleotides, 3.8 Å for polypeptides, and 5.0–5.3 Å for polysaccharides. Smaller units of 3.3 and 2.7 Å were measured for dsDNA and polyP, respectively. The second is the radial charge density around the polymer backbone (i.e., in the *yz* plane extending out from the backbone through the negatively charged substituents, shown in blue in Figure 2A), which is designated λ*yz* and computed according to the definition: λyz=(∑_i_q_i_/d^i^yz)/N where d^i^yz is the distance of each negative charge of the monomer from the main axis of the polyanion to the nearest negative charge in the *yz*-plane (see Figure 2A). Accordingly, λ*x* values are expected to decrease with increasing monomer size, whereas λ*yz* values are expected to increase when the charge on the monomers is located closer to the polymer backbone.

**Figure 2:**
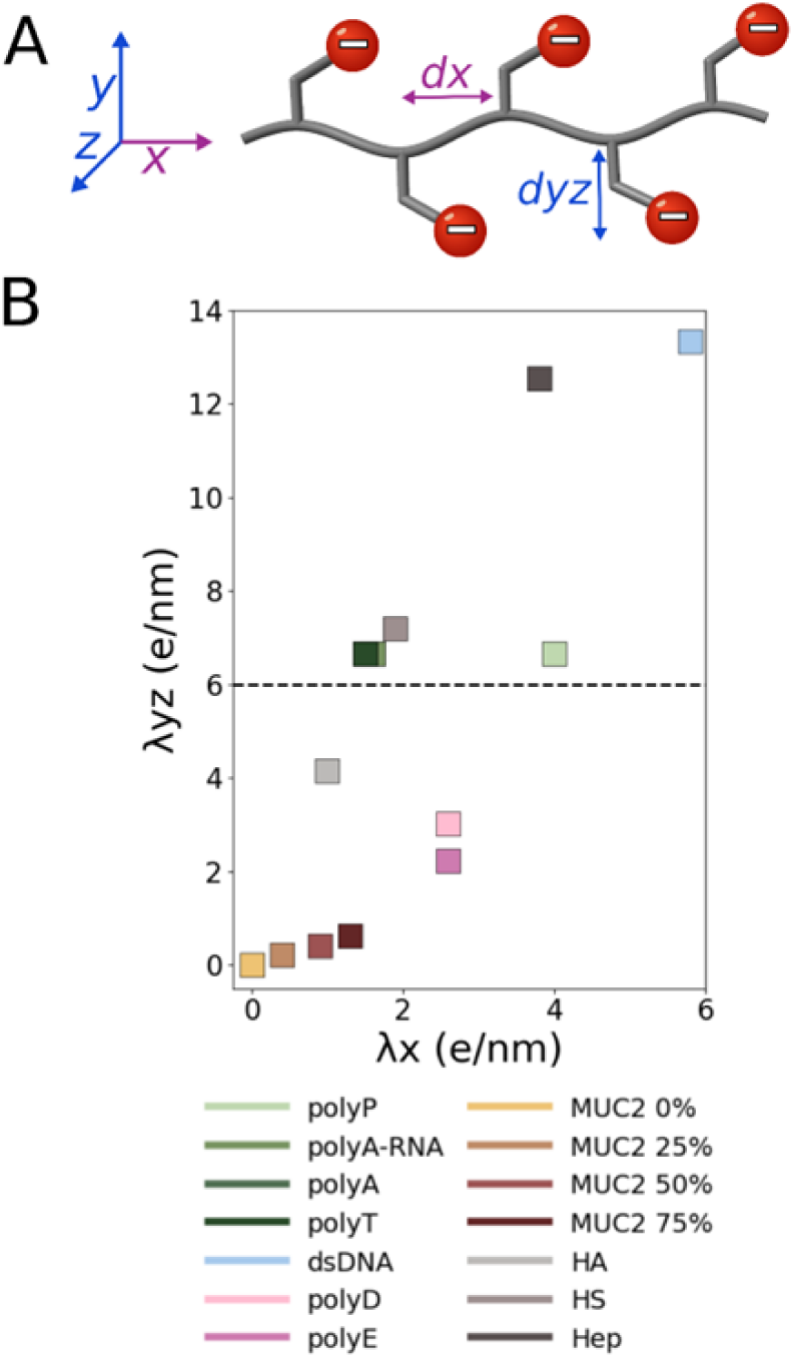
Charge distribution patterns in polyanions. (A) Schematic representation of the linear charge density (λ*x*) along the polymer backbone (*x*-axis, in purple) and the radial linear charge density (λ*yz*) in the plane perpendicular to the backbone (*yz*-plane, in blue). Monomer length (d*x*) is indicated in purple, and the radial distance of the negative charges from the backbone (d*yz*) is indicated in blue. Negative charges are denoted by red circles containing white minus signs. (B) Scatter plot of λ*x* versus λ*yz* values obtained for the polyanions. The points are colored according to the key under (A). Polyanions positioned below the dashed line (at λ*yz* = 6) have comparable λ*x* and λ*yz* values.

### Conformational Analysis and Flexibility of the Polyanions

Polyanion structural compactness was assessed by computing the end-to-end distance $\left(R_{ee}\right)$ of the polymers over time under the sodium and calcium regimes. In order to compare polyanions with different monomeric unit lengths, we normalized this value by each polyanion’s respective *L*_c_. To characterize the size measure of the polyanion chains, we considered the mean-squared end-to-end distance $<R_{ee}^{2}>$ and radius of gyration $<R_{g}^{2}>$ . The ratio of these two characteristic distances, sometimes called the size ratio, characterizes the average shape of a polymer chain.

To assess the flexibility of the polyanions, we performed principal component analysis (PCA) on the coordinates of anchor backbone atoms. PCA was carried out separately for polyanions sharing the same backbone architecture. Prior to the analysis, the atomic positions were superimposed, and the overall mean was removed, as implemented using scikit-learn.

### Sodium and Calcium Cation Interactions with the Polyanions

In order to understand cation organization around polyanions, we computed the radial density function (RDF) between the negatively charged oxygen atoms and the positively charged cations, using MDTraj along a radius of 3 nm and 200 bins. In addition, we performed a cation-binding analysis to quantify how tightly sodium and calcium cations interact with the polyanions. A cation was considered bound if it was located within 0.5 nm of a negatively charged oxygen atom. We further computed charge neutralization, a complementary method to assess cation binding.^60^ For each polyanion, we counted the number of negatively charged oxygen atoms within the 0.5 nm cutoff distance of any cation (using the same procedure as the binding analysis) and divided this value by the total number of negatively charged oxygen atoms. This yielded the fraction of charges neutralized. We further quantified cation bridging by identifying cases where a single cation was simultaneously within the 0.5 nm cutoff of two different negatively charged oxygens and dividing this count by the total number of neutralized charged sites. Finally, to investigate the solvent dynamics of the polyanions, we calculated the probability of cations in proximity to the polyanions within a cation–polyanion cutoff distance of 7.5 Å. The residence time of each of these cations was estimated by fitting their survival probability to an exponential decay function.

## Results and Discussion

### Polyanion Diversity in Charge Densities Suggests Distinct Biophysical Properties

To explore the role of polymeric properties in polyanion diversity,^61–64^ we first examined their negative charge distribution. We decomposed the charge distribution into two linear components: λ*x* and λ*yz*. The λ*x* component represents the linear charge density along the polymer backbone (*x*-axis; see Figure 2A in purple), whereas λ*yz* describes the radial linear charge density around the backbone along the orthogonal *yz*-plane (Figure 2A in blue). Plotting λ*x* against λ*yz* (Figure 2B) illustrates that the two charge densities are positively correlated, yet the correlation is not perfect, indicating that they indeed capture different properties of the polyanions.

Figure 2 shows that the three most densely charged polyanions (i.e., with the largest λ*x*) are polyP, Hep, and dsDNA (λ*x* ≈ 4–6 e/nm). Notably, Hep and dsDNA possess substantially higher λ*yz* values than polyP (Figure 2B). Although the charged groups in dsDNA and polyP are positioned at comparable radial distances from their backbones, one phosphoanhydride bond away in both cases, dsDNA exhibits a greater linear charge density and a markedly greater radial charge density compared with polyP, highlighting how polyanions sharing comparable backbone chemistry and linear charge density might exhibit distinct biological properties due to differences in radial charge density.

The second cluster of polyanions with high charge densities (λ*yz* ≈ 6.5–7.5 e/nm and λ*x* ≈ 2–4 e/nm) consists of the single-stranded polynucleotides polyA-RNA, polyA, and polyT, which display overlapping λ*x* and λ*yz* values; HS, whose λ*x* and λ*yz* values are slightly higher than those of the single-stranded polynucleotides; and the anionic polypeptides polyD and polyE, with the highest λ*x* but lowest λ*yz* values. These single-stranded polynucleotides have an identical radial distance and a similar number of charges compared with polyP, which leads to polyA-RNA, polyA, polyT, and polyP sharing similar λ*yz* values. However, polyP’s monomers are smaller than those of the other single-stranded polynucleotides, resulting in a similar amount of charge spread along a shorter distance, leading to polyP having a significantly higher λ*x* value, which is more than twice that of the other single-stranded polynucleotides. Unlike the single-stranded polynucleotides, the anionic polypeptides polyD and polyE present identical λ*x* values due to their having the same repeat unit size and total charge. The side chains of polyD are shorter than those of polyE, granting polyD a greater radial charge density than that of polyE.

Finally, the third cluster of polyanions with the lowest charge densities (λ*x* ≈ 0–2 e/nm) includes the MUC2 systems, which show a gradual increase in both λ*x* and λ*yz* with increasing levels of glycosylation, reflecting the addition of five charges at relatively long distances; and HA, which shares a similar λ*x* value as the highly glycosylated mucins but possesses a substantially higher λ*yz* value. Notably, MUC2 75% displays a marginally larger λ*x* than HA, despite having the same total number of charges, which can be attributed to HA having a larger average monomer size (5.0–5.3 Å), which exceeds that of the amino acid residues (3.8 Å). However, HA may compensate with charges positioned closer to the backbone, leading to its λ*yz* value being more than double that of MUC2 75%.

Interestingly, the polynucleotides and two of the polysaccharides (HS and Hep) possess λ*yz* values that are greater than their λ*x* values, which places them above the dashed line at λ*yz* = 6 e/nm. The λ*x* of dsDNA is approximately 3-fold greater than that of the single-stranded polynucleotides, due to its higher total charge and smaller unit size. Conversely, the polypeptides, MUC2 systems, and HA below the dashed line show similar λ*x* and λ*yz* values. Overall, these differences in linear and radial charge densities across polyanions suggest that even polyanions with similarity in terms of chemistry and structure can display distinct biophysical properties. Such distinctions may, therefore, offer insight into why diverse classes of polyanions, with tuned charge densities, may have evolved for different biological functions.

### Structural Characterization of Polyanions and Its Dependence on Cation Valency

To investigate how the differences in the linear and radial charge densities among polyanions might translate into distinct biophysical characteristics, we next quantified the compactness of each system using the end-to-end distance. To compare between polyanions that differ with respect to their monomer sizes, we normalized the end-to-end distance of each polyanion by its respective *L*_c_.^65,66^

We first compared *R*_ee_ distributions for each system under sodium and calcium ion conditions (Figure 3A). Interestingly, there seems to be a wider range of distributions under the Na^+^ condition, suggesting that it does not induce as powerful compaction as Ca^2+^. Indeed, the polyanions and the polypeptides, in particular, tend to exhibit, on average, lower end-to-end distance distributions for Ca^2+^ than Na^+^ (Figure 3A).

**Figure 3:**
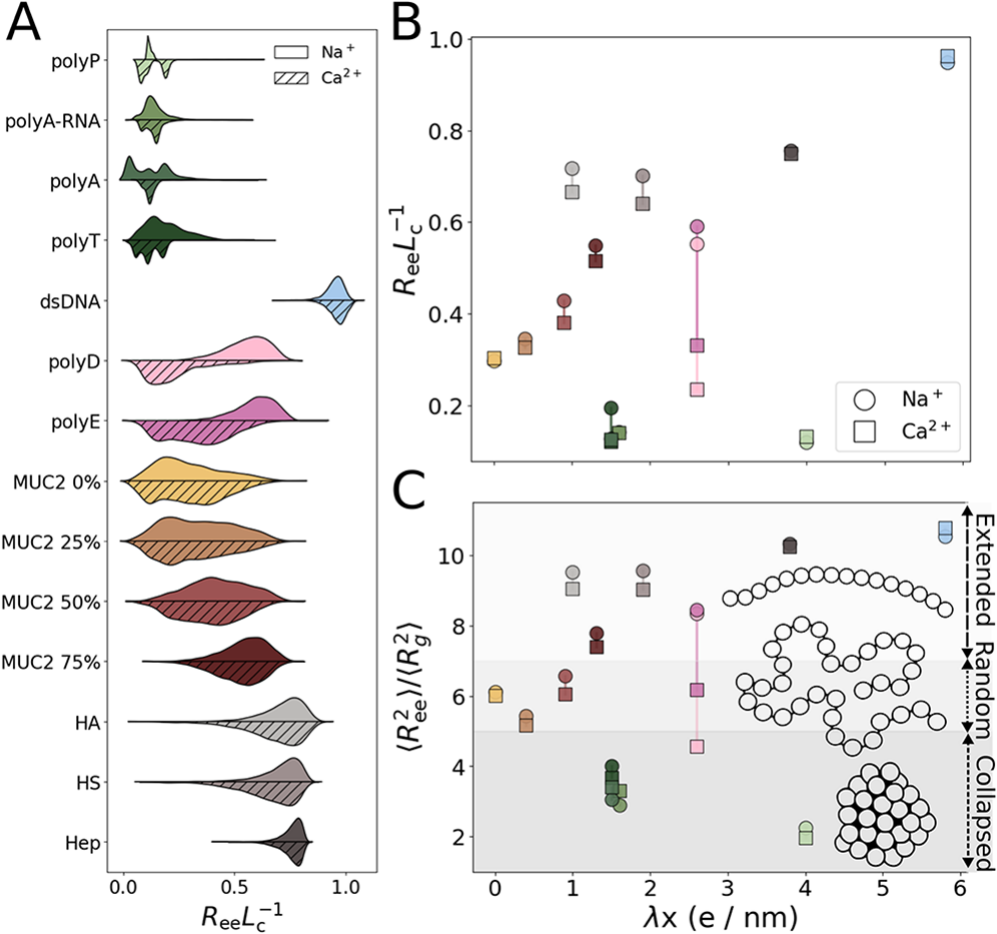
Structural characterization and flexibility of polyanions. (A) Violin plots of the distribution of the normalized end-to-end distance (indicating compactness) of the polyanions at ion concentrations of Na^+^ = 0.125 M (left, solid) and Ca^2+^ = 0.0625 M (right, hatched). (B) Scatter plot of the polyanions’ normalized end-to-end distance (i.e., *R*_ee_/*L*_c_) vs λ*x* under Na^+^ and Ca^2+^ conditions. (C) Scatter plot of the < *R*_ee_^2^>/<Rg^2^> vs λ*x* under Na^+^ and Ca^2+^ conditions.

We next examined the relationship between λ*x* and the normalized end-to-end distance to investigate how diversity in linear charge density modulates polyanion conformations (Figure 3B). Overall, we observe a positive linear correlation between *R*_ee_ and the linear charge density. For instance, polyanions that were found to have a higher charge density, such as Hep and dsDNA, also tended to display the strongest *R*_ee_ values (Figure 3B). The MUC2 systems also nicely follow this linear trend, with each glycosylation level incrementally increasing the *R*_ee_ values of the polyanion chain, suggesting expansion and swelling of the mucin chains as a function of the number of negatively charged groups. In contrast, the single-stranded polynucleotides, along with polyP, deviated from this trend (Figure 3B). These systems exhibited the lowest, and remarkably similar, *R*_ee_ values, indicating that under these conditions they preferentially adopt collapsed conformations despite their substantial linear charge densities. Examining the influence of Ca^2+^, we found that the polypeptides were the most sensitive group, showing the largest reductions in *R*_ee_ in the calcium condition. Overall, these analyses demonstrate that the polyanions collectively span nearly the full range of the *R*_ee_–λ*x* phase space, with different polyanion classes occupying characteristic regions. Notably, single-stranded polynucleotides and polyP behave distinctly from the other systems, reflecting how charge density alone does not fully determine the polymer conformation.

Furthermore, to characterize the preferred size measure of the polyanion chains, we considered the ratio between the mean-squared *R*_ee_ and *R*_g_ (Figure 3C). This metric provides a convenient indicator of the conformational regime adopted by each system.^66,67^ As expected, dsDNA and the polysaccharides (i.e., HA, HS, Hep) displayed the highest values for this ratio (∼10–12), consistent with extended conformations and rigid-rod-like properties. In contrast, the single-stranded polynucleotides and polyP displayed the lowest values (<4), typical of collapsed-like systems. The various MUC2 systems occupied an intermediate regime, with values typical of random coil-like properties (∼6). Finally, both the highly glycosylated MUC2 variant and the anionic polypeptides fell between the random-coil and rod-like limits, indicating partially extended but not fully extended chain properties.

Figure 4 shows snapshots of the conformations of the polyanions at the stated average *R*_g_ values under Na^+^ and Ca^2+^ conditions. The single-stranded polynucleotides, especially ssDNA and the polypeptides (polyD and polyE), undergo compaction under divalent cationic conditions, with a shrinkage of up to 0.8 nm (≈36% of the calculated expanded form for polyD, for example). The results under the sodium condition are in good agreement with the literature,^3^ as is the compaction of these groups with divalent ions.^68^

**Figure 4:**
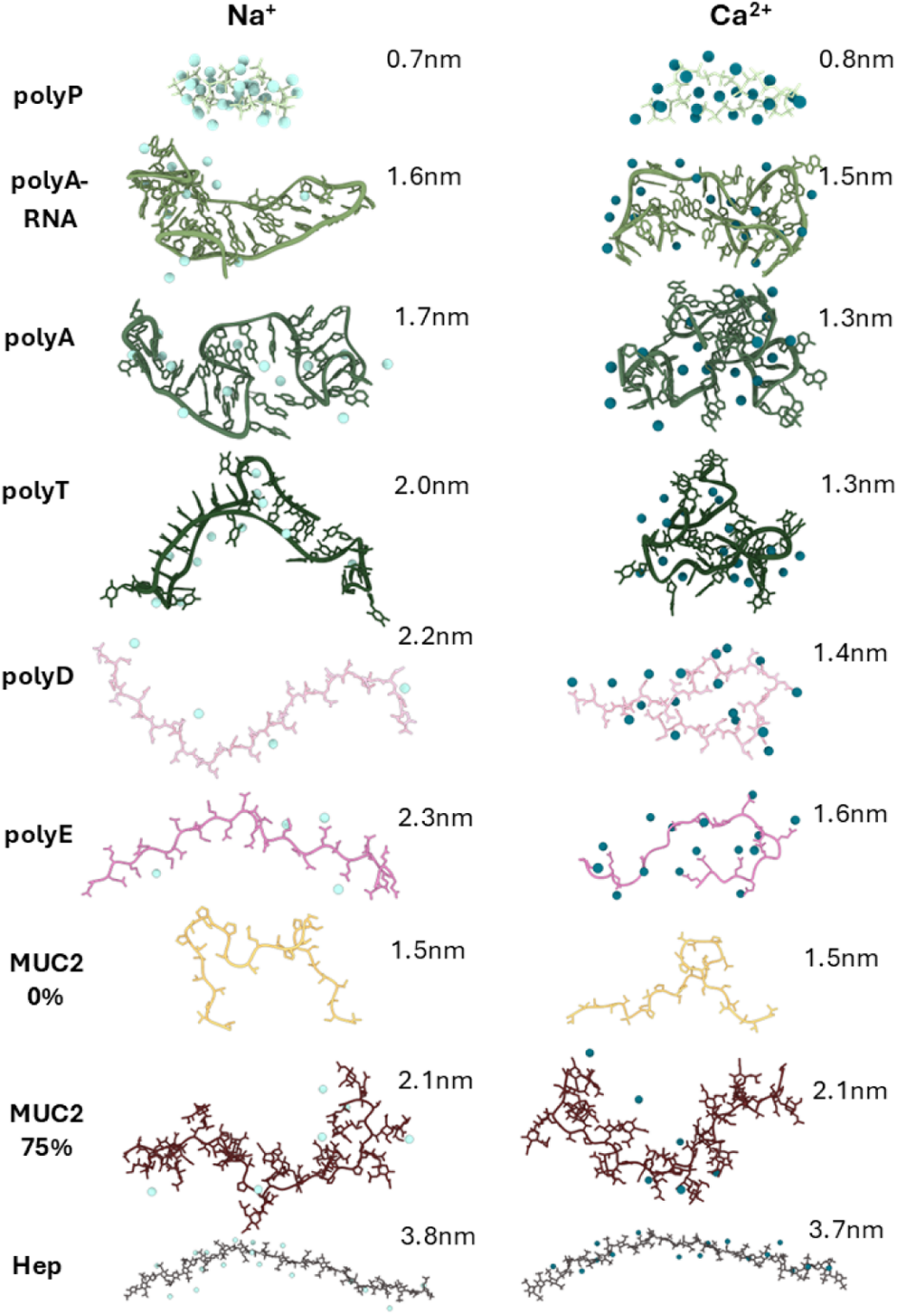
Structural impact of cation binding. The polyanion conformations were obtained under Na^+^ and Ca^2+^ conditions (left and right columns, respectively). The average radius of gyration (*R*_g_) of each polyanion is given to the right of each conformation. Only cations located within ≤0.75 nm of the polyanion were considered.

Together, these analyses show that polyanions differ markedly in their degree of compactness and that cations can modulate these properties in a system-specific manner. Some polymers are more sensitive to the presence of sodium or calcium than others, and their compactness adjusts accordingly. Notably, the polyanions collectively span the full range of conformational space defined by compactness and charge density, with each family of polyanions occupying a characteristic region of this space. Within each family, individual systems also display distinct properties, despite their underlying chemical similarities. For example, the MUC2 variants exhibit a clear decrease in compactness with increasing glycosylation, whereas the polysaccharides show minimal changes in compactness despite the increase in charge densities. These observations, on one hand, suggest that chemically related polyanions possess tunable biophysical properties that evolution could exploit to diversify biological function and, on the other hand, motivate us to examine backbone flexibility to investigate whether the intrinsic differences in charge density might influence other polyanionic properties.

### Flexibility and Dynamics of the Polyanions

To explore the conformational dynamics and flexibility of the polyanions, PCA was performed on the positions of anchor backbone atoms for each of the 11 studied polyanions (Figure 5). PCA was conducted separately for groups of polyanions sharing the same backbone architecture, with the anchor atoms for each group indicated by black circles in Figure 5A. Across all systems, more than half of the data variance is captured by the first two principal components. Overall, the PCA results align closely with the compactness analysis. Among the polypeptides, polyE exhibited the most restricted clustering in PCA space, consistent with a less flexible backbone, whereas polyD showed a slightly broader distribution, suggesting slightly greater flexibility, presumably due to ion-mediated interactions involving adjacent charges (Figure 4). A similar trend was observed in the MUC2 systems, where the spread of the PCA clusters increased with decreasing glycosylation, indicating reduced backbone flexibility as the number of negative charges increased. Moreover, dsDNA showed a markedly restricted positional distribution compared to the single-stranded polynucleotides, consistent with its high ⟨*R*_ee_^2^⟩/⟨*R*_g_^2^⟩ ratio, suggesting, as expected, rod-like properties and a rather inflexible backbone. PolyP also displayed a highly compact cluster in the PCA space, reflecting ion-driven packing interactions (Figure 4) that limit its accessible conformational space.

**Figure 5:**
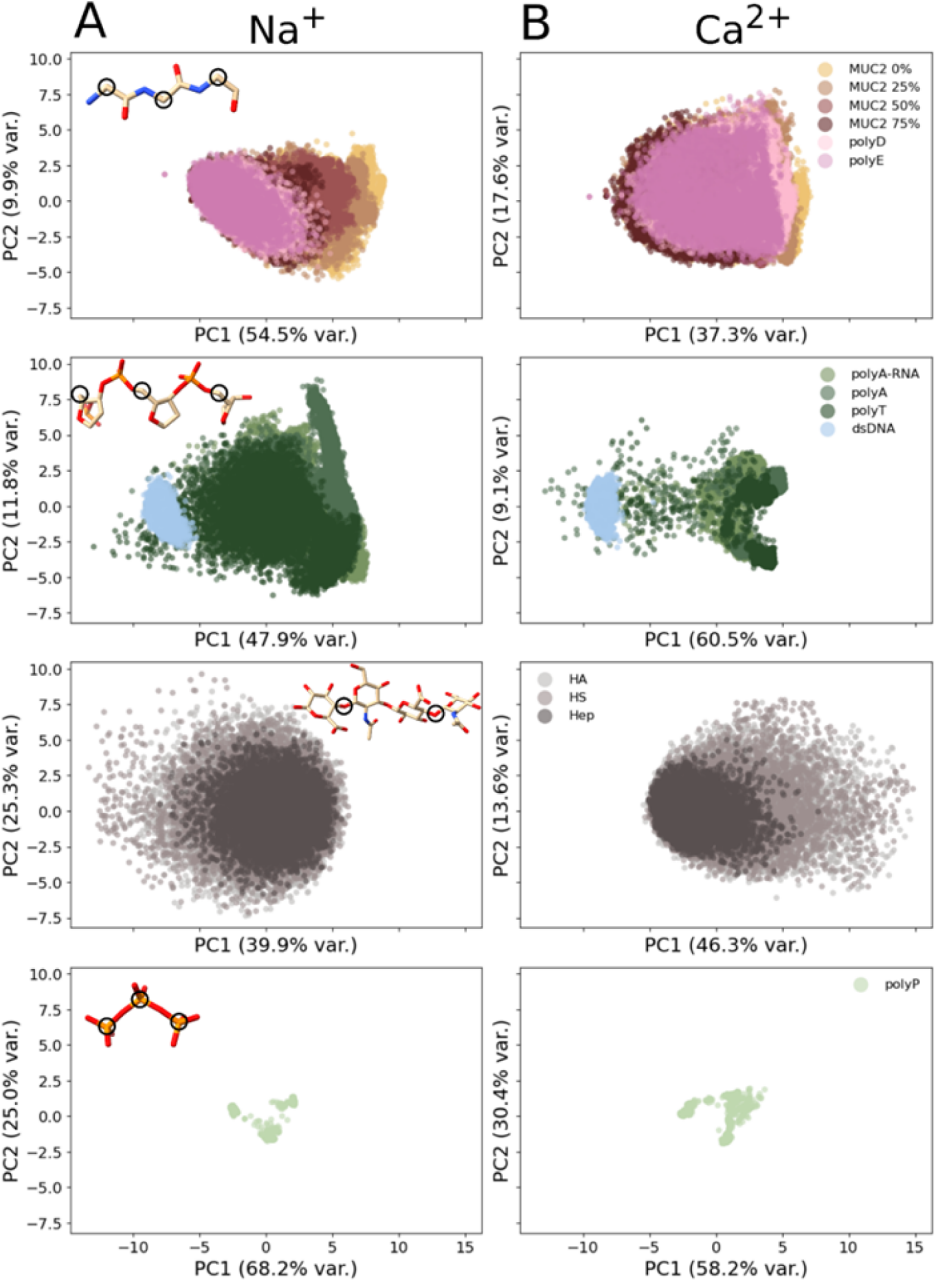
Principal component analysis (PCA) of polyanion backbone motions under Na^+^and Ca^2+^ conditions. Scatter plots of the first two principal components of the covariance matrix (PC1 and PC2) versus one another. Each system is represented by three replicates. The percentage of variance captured by PC1 and PC2 combined varies across systems and when simulated with Na^+^ (A) or with Ca^2+^ (B) and is indicated in parentheses. For comparison, the PCA was applied together for each type of polyanion that shares a similar backbone. The atoms that are used in each case to compare the conformational flexibility are circled in black on the molecules shown on the left.

Examining the effect of Ca^2+^, we found that calcium generally reduced the size of the PCA clusters across systems, indicating a decrease in backbone flexibility upon divalent ion–polyanion interactions. Interestingly, the single-stranded polynucleotide and polypeptide systems showed the highest number of Ca^2+^ interactions (Figures 6 and 7), and this was reflected in their PCA distributions, where single-stranded polynucleotides displayed a pronounced reduction in cluster size, whereas polypeptides exhibited more overlapping clusters, consistent with Ca^2+^ mediated constraints on their conformational space.

**Figure 6:**
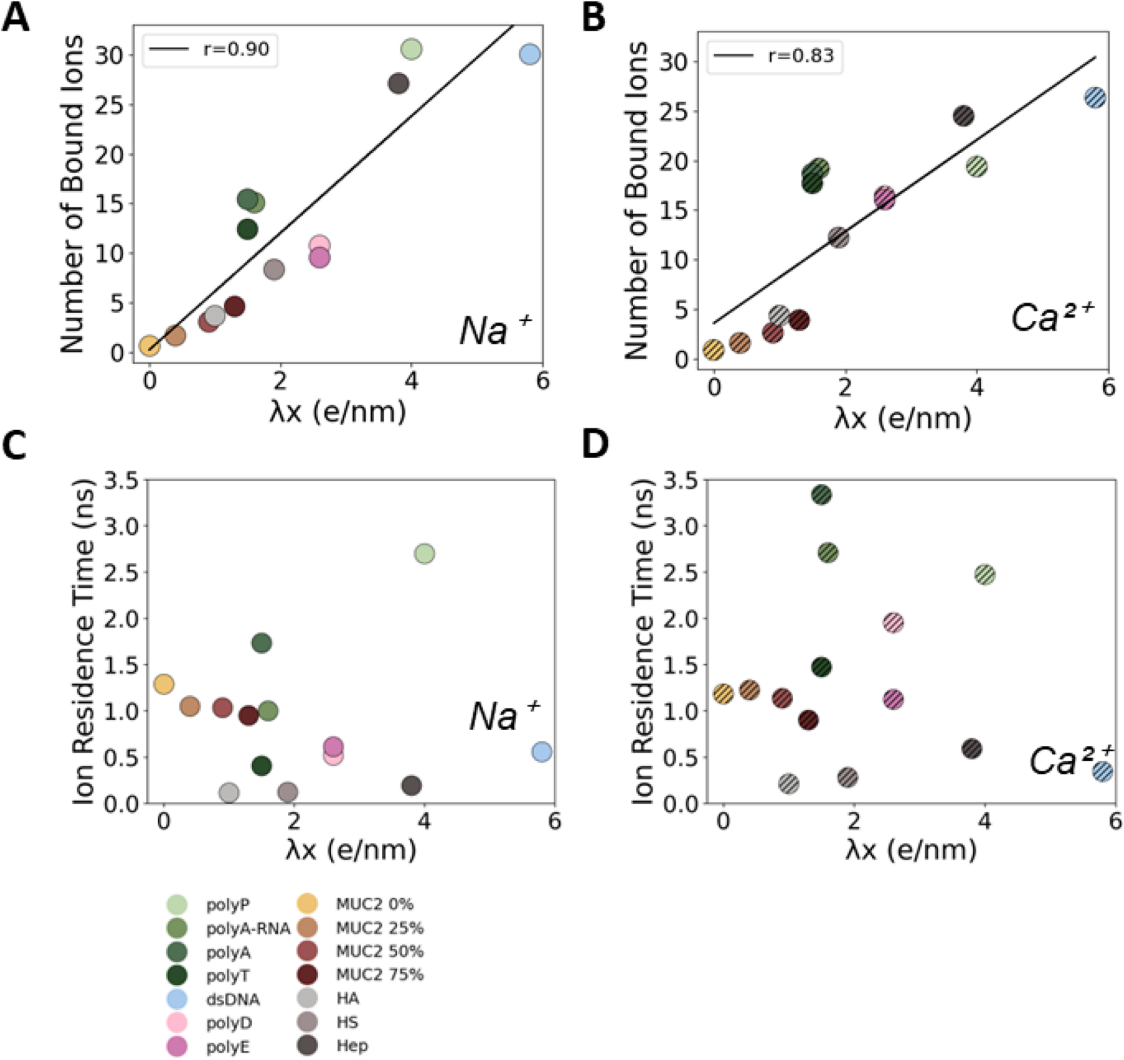
Cation binding to polyanions. Scatter plots depict the linear correlation (black lines) between the average number of bound cations (Na^+^ or Ca^2+^) and their linear charge density (λ*x*), where cation binding was defined as cation–polyanion distance ≤0.5 nm. Pearson’s correlation coefficients (*r*) are displayed in the top left corner of each plot. (A) and (B) show cation binding vs λ*x* for Na^+^ and Ca^2+^, respectively. (C) and (D) present scatter plots of the average cation residence time on the polyanions vs λ*x* for Na^+^ and Ca^2+^, respectively.

**Figure 7:**
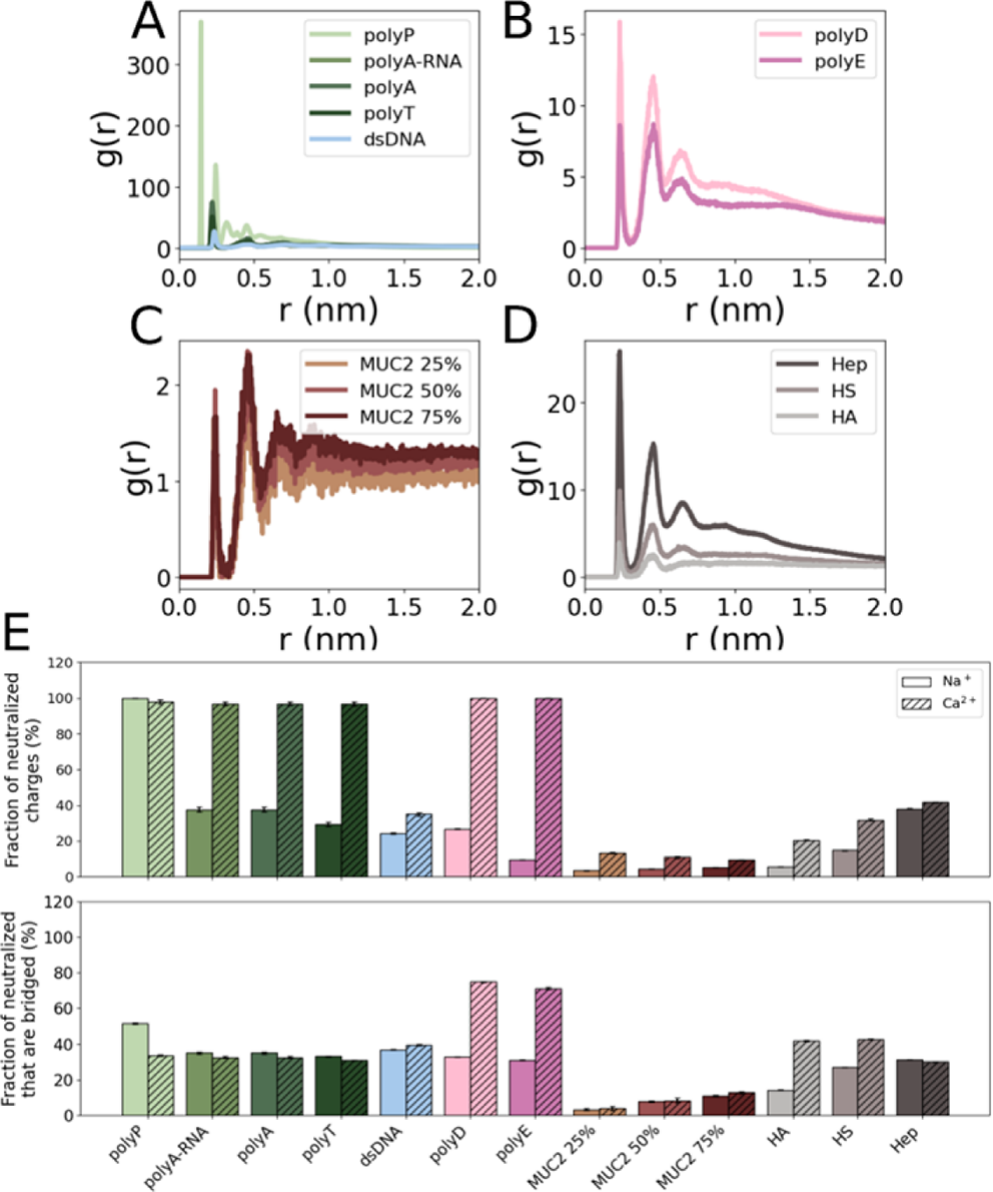
Radial distribution function (RDF) of Na^+^ around polyanions and its ability to neutralize polyanion charge. RDF of polyanionic: (A) polynucleotides, (B) polypeptides, (C) MUC2 systems (excluding 0% glycosylation), and (D) polysaccharides. These panels show three prominent peaks denoting sodium ion binding at distances of ∼0.25, 0.5, and 0.7 nm. (E) Top: Bar plot of the fraction of polyanion charges neutralized by cations within a cutoff distance of 0.5 nm. Error bars represent standard deviations. Bottom: Bar plot of the fraction of polyanion charges that are neutralized via a cation that neutralizes another negative charge. This measure depicts the percentage of cation-mediated interactions between two negative charges. The fraction of neutralized ions that are bridged is shown as a percentage of the total number of ions that are neutralized (upper panel).

The apparent similarity in motion across different polyanion types prompted us to investigate whether ion-binding dynamics and charge neutralization might play a more critical role in influencing their stability and function in biological environments.

### Cation Dynamics and Binding to Polyanions

We anticipated that polyanions with higher linear charge density would attract and bind more cations in a linear manner, with a preference for divalent cation binding, as their greater valency creates stronger electrostatic forces. To test this, we calculated the average number of mono- and divalent cations (Na^+^ and Ca^2+^, respectively) bound to the polyanions. These metal ions were chosen both for their prevalence in native solutions and for their similar apparent ionic radii (1.02 Å for Na^+^ and 1.00 Å for Ca^2+^).^69,70^ The divalent ion concentration was set to half that of the monovalent ion to maintain equal total charge for each system.

The λ*x* values generally correlate well with the number of bound sodium and calcium ions (Figure 6A and B). Accordingly, it may serve as a predictor of cation binding when comparing polyanions. In general, this illustrates that both mono- and divalent conditions have a strong preference for polyanionic molecules that possess a greater charge density.

The three most densely charged polyanions (polyP, dsDNA, and Hep) attract the most cations, whereas the MUC2 systems, which have the lowest linear charge density, attract the fewest (Figure 6A and B). Interestingly, polyP binds more sodium ions on average (31 ± 2; achieving total charge saturation) than do dsDNA and Hep (30 ± 3 and 27 ± 3, respectively), despite possessing a smaller total charge and comparable or lower λ*x* values. The lower monovalent binding of dsDNA and Hep, compared to polyP, may be due to the lower backbone flexibility. This structural rigidity likely not only hinders their ability to effectively displace ions but also prevents them from rearranging to allow optimal cation screening and packing, thereby reducing the number of cations able to bind at any given time. This may also explain why they cannot reach saturation, even under divalent conditions (Figure 7E).

The three most highly charged polyanions and MUC2 75% tend to bind more Na^+^ than Ca^2+^, however, this trend is reversed for the remaining polyanions. HA, HS, the single-stranded polynucleotides, and the polypeptides bind 1–7 more Ca^2+^ compared with Na^+^, with HA at the low end and polyE at the high end of this range. Moreover, dsDNA binds roughly twice as many Na^+^ as the single-stranded polynucleotides (with polyT binding less than half), but this difference diminishes for Ca^2+^. In contrast, MUC2 systems show a consistent linear increase in the number of cations (Na^+^ or Ca^2+^) bound with increasing λ*x*, with the total number of bound ions remaining low (≤5 ± 2) due to small λ*x* values. HA also falls within the low range (4 ± 2 for Na^+^ and Ca^2+^).

Ion dynamics are expected to be influenced by the charge density, but how this might occur has not been fully elucidated. Figures 6C and D show the ion residence times for Na^+^ and Ca^2+^ for each system. In general, the number of bound ions correlates with λ*x*. However, the relationship between cation residence time and λ*x* is more complex, with some of the polyanion groups exhibiting positive correlations to various extents but without a general relationship emerging. For instance, under the Ca^2+^ condition (Figure 6D), the sugars (HA, HS, and Hep) show a moderate positive trend of increasing cation residence times as λ*x* increases, whereas the MUC2 systems show a negative correlation (under both ionic conditions). The difference between polyD and polyE also becomes far more pronounced under calcium compared with sodium, with polyD showing an ∼1 ns longer residence time. In general, the peak residence times are lower under Na^+^ compared with Ca^2+^. These results indicate that it is cation valency rather than linear charge density that can influence cation residence times.

The single-stranded polynucleotides and polyP have the highest ion residence times. Among them, polyA has the highest standard deviation and exhibits the highest residence time, ∼3.4 ns under the calcium ion condition. Indeed, although the general overarching trend in this group of polyanions is that Ca^2+^ is associated with higher residence times, which is consistent with calcium’s higher valency granting it a higher binding affinity, the MUC2 systems and dsDNA do not follow this trend. Specifically, MUC2 systems show negligible differences in behavior between the different valencies, whereas dsDNA shows a slightly increased residence time under Na^+^. It is surprising that the addition of charges to MUC2 systems does not result in longer residence times, especially under calcium. However, greater charge density does not appear to equate to longer residence times for Hep or dsDNA either. Compared with Na^+^, the Ca^2+^ environment generally has a greater influence and increases the time spent near the polyanions, which again indicates that the capacity to influence cation residence times arises from valency rather than from the number of charges or charge density.

Given the trends described in the previous paragraphs, it is interesting to note that the three most densely charged polyanions do not have the highest ion residence times under all conditions. PolyP has the highest residence time under sodium conditions (∼2.75 ns) but is overtaken by the single-stranded polynucleotides polyA-RNA (∼3.4 ns) and polyA (∼2.75 ns) in the calcium ion environment. Hep and dsDNA have relatively low cation residence times compared to those of the other systems.

Although the overall trend matches our expectations, the number of Na^+^ bound to the polyanions is low, given that Na^+^ has half the valency of Ca^2+^. We expected an increased number of bound monovalent cations to offset the polyanionic charges. To better understand the distribution of cations around the negatively charged polyanion sites (i.e., around the acidic oxygens) relative to the bulk solution, the radial distribution function (RDF) was measured (see Figure 7A–D). This analysis allowed us both to quantify the likelihood of cation presence as a function of distance and to characterize cation arrangement for different polyanions, determining whether oxygen–cation binding was primarily direct or mediated by solvent interactions.

We compared the RDF both within and between systems: Figure 7A displays the polynucleotides, Figure 7B the polypeptides, Figure 7C the MUC2 systems (excluding 0% glycosylation, which lacks negative charges), and Figure 7D the polysaccharides. Focusing on regions within 2 nm of the polyanions enabled several hydration layers to be analyzed. All polyanions are characterized by three distinct peaks at approximately 0.25, 0.5, and 0.7 nm, followed by an asymptotic approach to *g*(*r*) = 1 at larger distances. This asymptotic behavior is expected, since *g*(*r*) is normalized to bulk water. The first peak, at ∼0.25 nm, corresponds to direct interaction and binding of the cations to the negative charges on the polyanions, where the filled orbital of the acidic oxygen interacts with the empty orbital of the metal cation.^71^ For most groups, it also has the highest peak (denoting the greatest probability of being found at that distance relative to the bulk solution). This peak is especially high for polyP, whose peak is more than three times the height of the next highest peaks (polyA and polyA-RNA). This finding is expected, as in all polyanions the most important interaction between cations and polynucleotides should occur around the phosphate group.^71^

The second peak, at ∼0.5 nm, captures the second layer of neighboring cations and usually has a lower probability than the first peak. The interaction is usually water-mediated.^71^ To quantify this behavior in our systems, we computed the hydration number for Hep and polyT, both of which display a secondary peak. For each of the three RDF peaks, the hydration number was calculated by counting the number of water molecules within a cutoff distance of 0.38 nm from Na^+^ ions assigned to that peak. We then considered the overall mean and standard deviation of these values. In both systems, we observe a progressive increase in hydration number from peak 1 to peak 2 and finally peak 3, with mean hydration numbers of 5.0 ± 1.1, 6.0 ± 1.3, and 6.2 ± 1.2 for Hep and 4.7 ± 1.6, 5.5 ± 1.6, and 5.8 ± 1.5 for polyT, reflecting the transition from direct contact to increasingly water-mediated ion interactions.

In four systems, namely, the three glycosylated MUC2 systems and polyE, the first peak is lower than or equal to the second peak. In MUC2 (Figure 7C), the first peak is lower than the second, showing that there is a greater probability of finding water-mediated ions at ∼0.5 nm, though their probability is lower overall than for the remaining polyanions. The peaks are of identical height in polyE (Figure 7B), indicating that there is an equal probability of finding Na^+^ ions in direct contact with the polypeptide at 0.25 nm and involved in water-mediated interactions at 0.5 nm. The RDF of polyE aligns qualitatively with the literature, which is likely because our analysis focused specifically on the negatively charged regions rather than the entire side chain.^72^ The presence of a third peak shows a longer-range order and a more complex interaction, with a much lower intensity than the two preceding peaks.^71^

In addition to their preference for a more distant, water-mediated interaction, MUC2 systems exhibit noticeable fluctuations in RDF profiles when compared with the other polyanions. This may derive from their lower charge density, as the weaker electrostatic interactions may encourage less stable binding, leading to fast “catch-and-release” binding. Alternatively, the glycoprotein may boost local viscosity, restricting cation movements and thus inhibiting smoother transitions in probability. It also appears that the distances for MUC2 are longer than those for other polyanions, suggesting their cutoff distance ought to be closer to ∼1 nm to adequately capture all the relevant bound ions.

To assess the efficiency of charge neutralization during these interactions, the fraction of charges neutralized in each polyanion was quantified (Figure 7E). The MUC2 systems consistently exhibit low levels of charge neutralization at a cutoff distance of 0.5 nm, with less than 10% of neutralized charges, on average, under the monovalent or divalent cation conditions, regardless of glycosylation percentage. Interestingly, although HA and MUC2 50% and 75% exhibit comparable levels of charge neutralization (<10%) in a Na^+^ environment, there is a 2-fold increase in charge neutralization for HA (∼23%) under a Ca^2+^ environment. The other systems also demonstrate significantly greater charge neutralization under calcium conditions (>35%), whereas under the sodium condition, less than half the amount of neutralization typically occurs. This is especially pronounced for the polynucleotides and other polypeptides (polyD and polyE), which show a much higher fraction of neutralized charges under Ca^2+^. However, polyP and Hep deviate from this pattern. In polyP, the large standard deviation obscures clear differentiation between Na^+^ and Ca^2+^, whereas in Hep, the difference is only slight.

We note that, under Ca^2+^, it is the single-stranded polynucleotides (ssDNA and ssRNA), rather than the most densely charged polyanions (i.e., polyP, Hep, and dsDNA), that have the highest fraction of neutralized charges. The polyP system follows closely behind, whereas dsDNA and Hep lag in comparison, probably because their inherent lack of flexibility reduces their capacity to attract surrounding ions, thus aligning them with the polysaccharides, which also display greater charge neutralization under Ca^2+^, albeit to a lesser degree.

When examining the fraction of neutralized charges bridged by the same cation (Figure 7E), we found generally moderate differences within most systems. In many cases, about 30% of negative charges interact with another negative charge via a bridging cation. Notable exceptions were the polypeptides, HA, and HS, all of which showed a clear increase in cation bridging under calcium conditions compared with sodium. The greater number of ion-mediated interactions that bridge two negative charges can explain the significant compaction of polyD and polyE in the presence of Ca^2+^ compared to that in Na^+^. This can explain the involvement of polyD and polyE and biomineralization.^73^ PolyP is the only system that showed the opposite trend, showing more cation bridging under sodium conditions compared with calcium.

Overall, for most polyanions, the charge neutralization data show that divalent cations neutralize a larger fraction of polyanion charge compared with monovalent cations. Furthermore, consistent with the conclusions drawn from the earlier cation residence time analysis, this effect is driven more by cation valency than by charge density. Consequently, polyanions possessing a larger charge density do not necessarily achieve better neutralization. This confirms that, for the purposes of achieving charge neutralization, steric limitations and molecular flexibility are more important than charge screening and cation binding.

## Conclusions

The current research aims to quantify and clarify differences among endogenous polyanions by examining how their molecular structure, charge distribution, conformational flexibility, and associated ions influence their biological activity. Ions are well known to contribute to the structure and function of many polyanions. For example, divalent ions play a critical role in DNA condensation.^74–76^ Similarly, mucin proteins respond differently to sodium and calcium ions; calcium promotes mucin compaction,^77,78^ and its concentration has been linked to disease states.^79,80^ Through a systematic investigation of multiple polyanions, we aim to map the landscape of their polymeric properties and characterize the nature of their interactions with ions, thereby shedding light on why biological systems employ such a diverse repertoire of polyanions.

Polyanions’ intrinsic differences in monomer size, charge distribution, and backbone architecture reflect the broad diversity of linear and radial charge densities observed in polyanions. Densely charged systems such as polyP, Hep, and dsDNA share high λ*x* values but differ markedly in λ*yz*. Single-stranded polynucleotides, polysaccharides, and anionic polypeptides occupy intermediate charge density regimes, while MUC2 variants and HA display the lowest charge densities, with glycosylation modulating λ*x* and λ*yz* in predictable increments. These patterns indicate that polyanions with similar chemistry and backbone structure can nonetheless exhibit distinct biophysical properties, suggesting a pathway for the evolutionary diversification of polyanionic functions.

These differences in intrinsic properties translate into differences in compactness, flexibility, and ion interactions. End-to-end as well as mean-squared *R*_ee_ and *R*_g_ ratio analyses show that polyanions span the full conformational landscape, from rigid, rod-like chains (dsDNA, Hep, HA) to collapsed states (single-stranded polynucleotides, polyP), with MUC2 variants preferentially adopting an intermediate random-coil regime. Cation valency modulates these conformation preferences, with Ca^2+^ inducing compaction across most systems, particularly polypeptides and single-stranded polynucleotides, and reducing backbone flexibility, as reflected in PCA analyses.

Ion binding correlates well with λ*x*, making linear charge density a useful predictor of cation–polyanion association, with both monovalent and divalent ions preferentially binding to more densely charged polyanions. Ion residence times, however, show more complex behavior, depending primarily on cation valency rather than charge density. Divalent cations consistently neutralize a larger fraction of charges and tend to promote more bridging interactions, potentially explaining the Ca^2+^mediated compaction observed in several systems.

In conclusion, using all-atom molecular dynamics simulations of 11 representative polyanions spanning three major classes of polyanionic biomacromolecules, this study shows that the various biological polyanions have different intrinsic molecular properties (e.g., monomer size, structure, and charge density per monomer) that control their biophysical properties and, therefore, their interactions. We conjecture that, from an evolutionary perspective, the usage of diverse types of polyanions with varied polymeric and biophysical properties is essential to support different biological functions. It remains an open question as to why polycations are less common than polyanions.
